# Supplementary material for: Cryo-EM structures of the E. coli Ton and Tol motor complexes
Source: Nat Commun. 2025 Jul 1;16:5506. doi: 10.1038/s41467-025-61286-z (PMC12215075; doi:10.1038/s41467-025-61286-z)
Supplement: Supplementary file 2 — Description of Additional Supplementary Information [file 41467_2025_61286_MOESM2_ESM.pdf]

Description of Additional Supplementary Files

File Name: Supplementary Movie 1
Description: Structural variability of the TolAQR complex.
